# ZnO Nanorods Coated Single-Mode–Multimode–Single-Mode Optical Fiber Sensor for VOC Biomarker Detection

**DOI:** 10.3390/s22166273

**Published:** 2022-08-20

**Authors:** Kankan Swargiary, Prattakorn Metem, Chayapol Kulatumyotin, Suphavit Thaneerat, Noppasin Ajchareeyasoontorn, Pannathorn Jitpratak, Tanujjal Bora, Waleed S. Mohammed, Joydeep Dutta, Charusluk Viphavakit

**Affiliations:** 1 International School of Engineering (ISE), Intelligent Control Automation of Process Systems Research Unit, Chulalongkorn University, Bangkok 10330, Thailand; 2 Functional NanoMaterials Group, Department of Applied Physics, KTH Royal Institute of Technology, Hannes Alfvéns väg 12, 11419 Stockholm, Sweden; 3 Biomedical Engineering Program, Faculty of Engineering, Chulalongkorn University, Bangkok 10330, Thailand; 4 Center of Excellence in Nanotechnology, Asian Institute of Technology, Pathumthani 12120, Thailand; 5 Center of Research in Optoelectronics, Communication and Control Systems (BU-CROCCS), School of Engineering, Bangkok University, Pathumthani 12120, Thailand

**Keywords:** optical fiber sensor, surface functionalization, ZnO nanorod, volatile organic compound, biomarker detection

## Abstract

This work demonstrated a ZnO-coated optical fiber sensor for the detection of a volatile organic compound (VOC) biomarker for diabetes for detecting isopropanol (IPA) markers. A coreless silica fiber (CSF) was connected to a single-mode fiber (SMF) at both ends to achieve a SMF–CSF–SMF structure. CSF is the sensing region where multimode interference (MMI) generates higher light interaction at the interface between the fiber and sensing medium, leading to enhanced sensitivity. Optimization of the CSF length was conducted numerically to attain the highest possible coupling efficiency at the output. Surface functionalization was achieved via hydrothermal growth of ZnO nanorods directly onto the CSF at low temperatures. The optical fiber-based sensor was successfully fabricated and tested with 20%, 40%, 60%, 80%, and 100% of IPA. The sensor response was recorded using an optical spectrometer and analyzed for sensor sensitivity. The fabricated sensor shows the potential to detect isopropanol with the sensitivity of 0.053 nm/%IPA vapor. Further improvement of the sensor sensitivity and selectivity is also proposed for future work.

## 1. Introduction

Volatile organic compounds or VOCs are typically present as gases in air at certain temperature and pressure conditions [1]. VOCs are generally emitted to the atmosphere from household product ingredients, paints, wax, fuels, personal care products, and industries [2]. They can cause an adverse health effects [3] even at very low concentrations (ppb levels) [4]. Moreover, VOC is also emitted from the exhaled breath of a person when transported to the excretion organs via body fluids [5]. Concentrations of VOCs in exhaled breath are reported to be associated with several chronic diseases and can serve as viable biomarkers. For instance, acetone and isopropanol have been widely considered as biomarkers for type 1 diabetes [6] and lung cancer, respectively [7].

There are various applications for sensing VOCs that lead to a lot of interest for their monitoring, detection, and analysis. Spectrophotometry [8], gas chromatography [9], and high-performance liquid chromatography [10] are often employed for the accurate determination of VOCs. These conventional methods are generally time consuming, expensive, and require highly skilled personnel for operation. Therefore, cost-effective and portable gas-sensing devices are essential [11]. A variety of methods have been explored based on different sensing principles such as electrochemical, optical, and resistive. Electrical methods focus on measurements of changes in resistances and potentials in the presence of target VOCs [12,13]. Optical sensors involve light absorption, transmission, and intensity variations measured in a spectrometer [14,15,16,17,18].

Optical fiber sensors have been in consideration due to their advantages of being light weight, capable of remote sensing, being less prone to electrical noises, and multiplexing [14,15]. VOCs in breath are found in low concentrations of ppm to ppb levels. Optical fibers can be used as a sensor to detect these compounds. In particular, single-mode (SM)–multimode (MM)–single-mode (SM) or (SMS) fiber structure has been widely explored for many biomedical applications. Utilizing the coreless silica fiber (CSF) at the multimode fiber (MMF) region helps in improving the light interaction with the surrounding environment. This CSF fragment acts as a sensing region of the sensor for achieving the maximum possible sensitivity. Moreover, the fabrication process of the SMS fiber-based sensor is straightforward, which is attractive for practical applications [19].

Recently, focus has been given to the surface functionalization mechanism at the sensing region of the optical fiber to improve its sensitivity and selectivity for VOC detection. As such, zinc oxide (ZnO) is a promising candidate that possesses many distinct advantages, such as high stability, wide band gap, and biocompatibility [20]. With these advantages, ZnO has a wide application in optoelectronics, nano sensing, and energy harvesting [21]. ZnO-coated optical fiber sensors are reported to have high sensitivity and selectivity for ammonium gas and ethanol [22,23]. In addition, sensors coated with ZnO nanostructure have been reported not to be affected by humidity, which makes them good candidates for the breath analysis as humidity is omnipresent in exhaled breath. For increasing the surface area of the ZnO coatings, nanostructures of nanoparticles or nanorods on the optical fiber are often used. Nanorods have been shown to further assist in guiding light due to refractive index differences [24]. ZnO nanorods have a higher refractive index (~2) compared with the silica fiber core (~1.5) that allow light coupling into nanorod waveguides by scattering light [24]. This gives rise to higher sensitivity of interaction between the guided light and the surrounding environment [25]. ZnO was also reported to have been utilized for monitoring and detection of refractive index [26], humidity [26,27], temperature [28], and different gases [29]. Subramaniyam et al. developed a fiber optic sensor based on pristine and amine functionalized ZnO flakes for detecting different VOCs [29]. Azad et al. reported a hydrothermally grown ZnO nanorod over the surface of the fiber as a humidity sensor by controlling the density of nanorods and the response time of the sensor [30]. These well-known effects of surface functionalization, when combined with SMS optical fiber structure, have attracted researchers to design a type of a sensor that involves this operation [24].

In this work, we extended our previous experience in hydrothermally grown ZnO nanorods coated onto the surface of the SMS optical fiber sensor for VOC biomarkers detection in breath. Isopropanol (IPA) vapor was considered as the VOC marker in this work as it is one of the biomarkers for non-invasive diabetes diagnosis [6]. IPA vapors with different concentrations ranging from 20% to 100% were obtained by the vaporization of IPA solutions in deionized (DI) water. The different concentrations of IPA solutions were left to evaporate at room temperature inside the chamber during the experiments. The length of the sensor was optimized by numerical modeling to maximize the sensitivity of the devices. The growth of ZnO nanorods onto the fiber itself with fabrication optimization and characterization of the sensor was studied. Finally, the obtained results from the fabricated device showed a good sensitivity and optical response to the VOC marker (IPA), which is promising for the future development of non-invasive diabetes monitoring systems.

## 2. Materials and Methodss

### 2.1. Materials and Reagents

SMF 28 was used as a single-mode fiber (SMF). Coreless silica fiber (CSF) with a diameter of 125 µm was used as a multimode fiber (MMF) region in the SMS optical fiber sensor structure. Sumitomo Electric Z2C core alignment fusion splicer and Sumitomo Electric FC-6S fiber cleaver were used for fiber assembly. For the hydrothermal growth process of ZnO nanorods, zinc acetate dihydrate (Merck KGaA, Darmstadt, Germany, CAS number 5970-45-6), zinc nitrate hexahydrate [Zn (NO_3_)_2._6H_2_O] (Ajax Finechem Pty Ltd., New South Wales, Australia, CAS number 10196-18-6) and hexamethylenetetramine or HMT [(CH_2_)_6_N_4_] (Sigma-Aldrich, Bangkok, Thailand, CAS number 100-97-0) were used. All the chemicals were of analytical grade and used without further purification. Details of the hydrothermal growth process can be found in our previous reports [31,32].

### 2.2. Sensor Design and Concept

**Figure 1:**
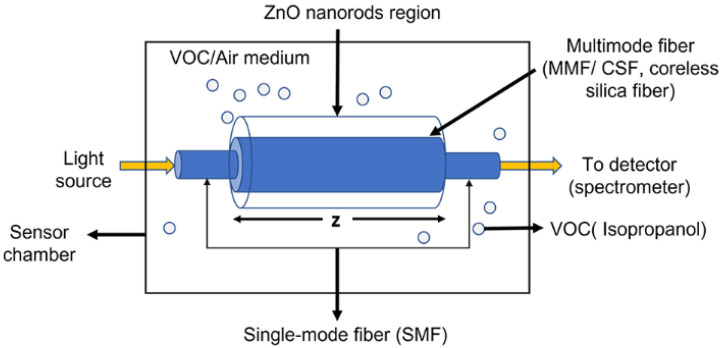
Schematic illustration of the designed sensor with ZnO nanorods coated on the CSF inserted between two SMFs.

The concept of the sensor design is schematically represented in Figure 1, which is based on single-mode–multimode–single-mode (SMS) fiber structure. The single-mode fiber acts as a waveguide for delivering light from the source to the sensing region and then to the detector, which is the spectrometer. It is utilized for recording the intensity spectra. On the other hand, the ZnO nanorods-coated multimode fiber acts as a sensing region. The multimode fiber (MMF) or coreless silica fiber (CSF) allows higher interaction of light at the sensor surface. This occurs due to the removal of the cladding region and hence we achieve a small index contrast between the fiber and the surrounding medium that allows a larger number of modes propagating inside the fiber compared with standard MMFs. The removal of the cladding and the large number of modes allow a higher intensity of the evanescent field at the fiber interface and hence increase the light–matter interaction between the evanescent field and the sensed material [33], leading to the enhancement of sensitivity. When the sensor is exposed to different environments such as in the presence of VOCs (IPA, in our case), the refractive index of the medium is affected, leading to a change in propagation constants and modal property inside the fiber. Hence, the multimode interference (MMI) spectrum is altered.

These changes in the spectrum can be recorded in an optical spectrometer and the data can be processed to correlate the shift due to the changes in the environment. The sensor region is coated with ZnO nanorods which were prepared by a green fabrication process in aqueous media to further enhance the sensitivity of the sensor through light scattering and increasing the light matter interaction region [34].

### 2.3. Numerical Modelling

**Figure 2:**
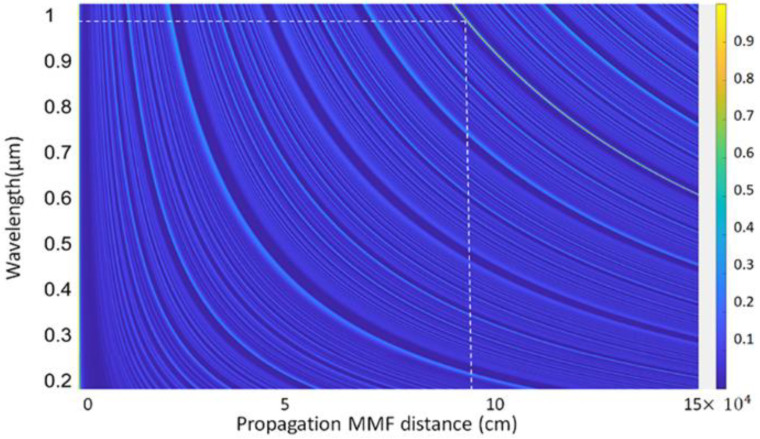
Coupling efficiency of the propagating distance of the CSF segment.

It is imperative to study the modal properties of the system, including the nanorods and their effect on multimode interference (MMI) and the reimaging distance before the fabrication of the SMS optical fiber structure. This was carried out theoretically using a similar model as that reported in our previous work [35]. The guided mode inside the SMF acts as the input field for the CSF segment. The field distribution at the end of the CSF segment of length z is written in Equation (1) [33],

(1) $$E_{MMF}\left(r,\theta ,z\right)\overset{M}{\underset{j=0}{\sum }}a_{j}\psi_{j}\left(r,\theta \right)\exp\left(i\beta z\right)$$

where $\psi_{j}$ and $a_{j}$ are the *j*th mode electric field amplitude and the field expansion coefficient, respectively. The constant *M* is the total number of guided radial modes located inside the CSF. Equation (1) represents the MMI of the different excited modes while propagating inside the MMF segment. The field at output of the MMF couples to the output SMF fiber. The output intensity of the SMS fiber is defined by the coupling efficiency, η [32] which is given in Equation (2),

(2) $$\eta =\overset{M-1}{\underset{j=0}{\sum }}\overset{M-1}{\underset{h=0}{\sum }}{\tilde{a}}_{j}{}^{2}{\tilde{a}}_{h}{}^{*2}\exp\left[i\left(\beta_{j}-\beta_{h}\right)z\right]$$

where ${\tilde{a}}_{j}$ is the modified expansion coefficient and $a_{j}$ is the expansion coefficient of the *j*th propagation mode is given by,

(3) $${\tilde{a}}_{j}=a_{j}\sqrt{\frac{P_{j,h}}{P_{s}}}$$

(4) $$a_{j,h}=\frac{\int_{\theta =0}^{2\pi }\int_{r=0}^{\infty }E_{s}\left(r,\theta \right)\times R_{j,n}{\left(r,\theta \right)}^{*}rdrd\theta }{P_{j,h}}$$

(5) $$P_{s}=\overset{2\pi }{\underset{\theta =0}{\int }}\overset{\infty }{\underset{r=0}{\int }}{\left|E_{s}\left(r,\theta \right)\right|}^{2}rdrd\theta$$

(6) $$P_{j,h}=\overset{2\pi }{\underset{\theta =0}{\int }}\overset{\infty }{\underset{r=0}{\int }}{\left|R_{j,h}\left(r,\theta \right)\right|}^{2}\;rdrd\theta$$

where j and h are the indices of the guided radial modes inside the MMF segment. Maximum coupling efficiency is obtained at the reimaging distance where the field EMMF resembles the input field and matches that mode of the output SMF fiber. The coupling efficiency (η) at a certain propagation length of the CSF over the broad wavelength is shown in Figure 2.

From Figure 2, the reimaging distance, at which maximum coupling efficiency was achieved, was 9.5 cm for a volume fraction of ZnO of 0.05 around λ = 958–980 nm. To achieve the maximum possible sensitivity, a fiber sensor with a length of 9.5 cm was fabricated for developing the actual device.

### 2.4. Sensor Fabrication

**Figure 3:**
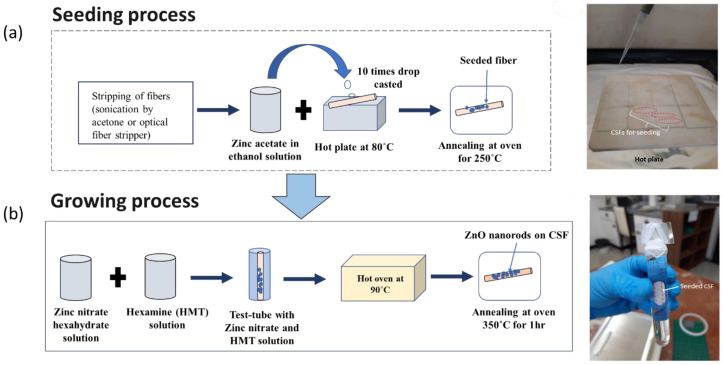
(**a**) ZnO seeding process showing the drop-casting of zinc acetate in ethanol onto heated CSFs; (**b**) growth of the ZnO nanorod process, showing the setup made of glass slides for suspending CSFs in the zinc nitrate hexahydrate and hexamine solution.

The proposed SMS optical fiber sensor was fabricated and the ZnO nanorods were coated onto CSF region by the hydrothermal method [34,36]. Firstly, the 125 µm diameter CSFs were cut into ~9.5 cm lengths and the fiber jacket was removed using an optical fiber stripper. Then, the seeding process was performed by placing the fiber samples on a hotplate at 80 °C followed by drop-casting zinc acetate dihydrate (Zn(CH_3_COO)_2_·2H_2_O) in ethanol (1 mM) solution onto the samples 10 times. Then, the fiber samples were annealed at 250 °C for 1 h in an atmospheric oven to reduce oxygen vacancies in the crystals, thus reducing electronic defects [37]. After completing the seeding process, the growth of ZnO nanorods was performed using equimolar solutions (10 mM) of zinc nitrate (Zn(NO_3_)_2_·6H_2_O) and hexamethylenetetramine (C_6_H_12_N_4_) in deionized water. This solution was then poured in a recipient with the fiber samples placed vertically and the recipient together with the fiber samples were put in an oven at 90 °C for growth of ZnO nanorods for 3, 4, 5, and 7 h, respectively. After the whole process was completed, the fiber samples were thoroughly rinsed with DI water and annealed at 350 °C for 1 h in an oven. The fabrication process involved is shown schematically along with the actual fabrication images in Figure 3a,b.

**Figure 4:**
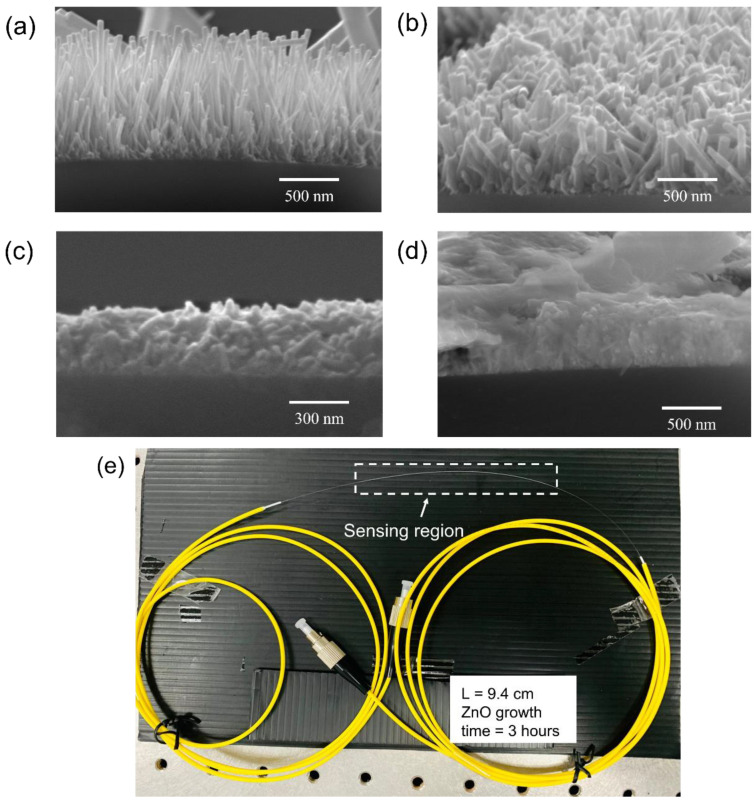
SEM micrographs of ZnO nanorods on CSF with growth times of (**a**) 3 h; (**b**) 4 h; (**c**) 5 h; and (**d**) 7 h, and (**e**) fabricated sensor with the sensing length of 9.4 cm.

The morphology of CSF samples was studied using SEM (Quanta FE SEM) and selected micrographs are shown in Figure 4a–d. Within a growth time of 3 h, ZnO nanorods were observed on the surface of CSF. For a growth time of 4–5 h, non-uniform secondary growth was observed [38,39]. Finally, after 7 h, a dense growth of the ZnO nanorods was noticed. Moreover, the longer time of hydrothermal growth of the nanorods resulted in rendering the fibers excessively fragile for splicing operation. Thus, 3 h of growth time of nanorods was considered to be the optimum for coating the SMS optical fiber.

Hence, the SMS optical fiber sensor with the ZnO nanorod hydrothermally grown for 3 h on a 9.4 cm-long CSF was fabricated. The CSF segments were affixed with the two SMF segments. The fabricated SMS optical fiber sensor is shown in Figure 4e in which the sensing region is highlighted in the drawn rectangle. The sensing region, which is the CSF coated with ZnO nanorods, was fused with the yellow patch optical fiber at both ends by a splicer (Sumitomo Electric, Z2C, Ozaka, Japan). The patch optical fiber is a SMF with FC/PC connector for the light source and spectrometer connection.

### 2.5. Experimental Setup

**Figure 5:**
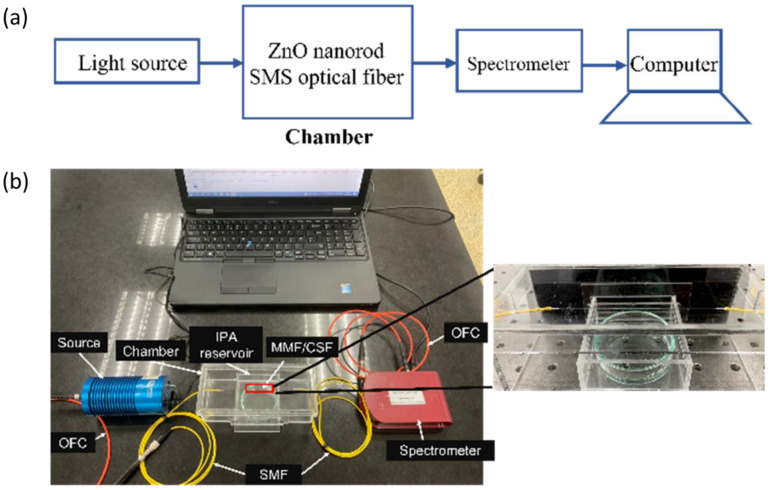
(**a**) Schematic representation of the experimental setup for characterizing the fabricated sensor; (**b**) actual image of an experimental setup.

The experimental setup used in this work is shown in Figure 5a,b. The setup consists of a visible light source (Ocean Optics HL-2000, Dunedin, FL, USA), optical fiber cable (OFC), sensor chamber, and a spectrometer (Compact CCD Spectrometers CCS200/M, Thorlabs, NJ, USA). The sensor chamber was made from 3 mm-thick acrylic polymer. It was designed in 3D printing software (Fusion 360) with an overall volume of 560 cm^3^. It was then fabricated using a laser cutting machine and assembled using acrylic mate. The IPA reservoir was designed to be underneath the sensor element to be directly exposed to the IPA vapor as shown in Figure 5b. In the presence of the IPA vapor, the surrounding refractive index inside the chamber changed, resulting in the progressive variation of optical spectra that were monitored by the spectrometer and recorded in a computer.

In the experiment, the intensity spectra of the fiber sensor exposed to water vapor (0% IPA) served as a reference ($I_{0}$). Then, the sensor was exposed with IPA vapor of different concentrations ranging between 20% and 100%. The intensity spectra were recorded every minute for 15 min after closing the chamber. This experiment was carried out at room temperature. After the experiment, nitrogen gas (N_2_) purging of the chamber was carried out to remove the IPA absorbed on the ZnO-coated sensing element and in the test container prior to any other measurements. The experiment was repeated with different IPA concentrations at least three times to investigate the repeatability and consistency of the fiber sensor.

### 2.6. Isopropanol (IPA) Vapor Preparation

**Figure 6:**
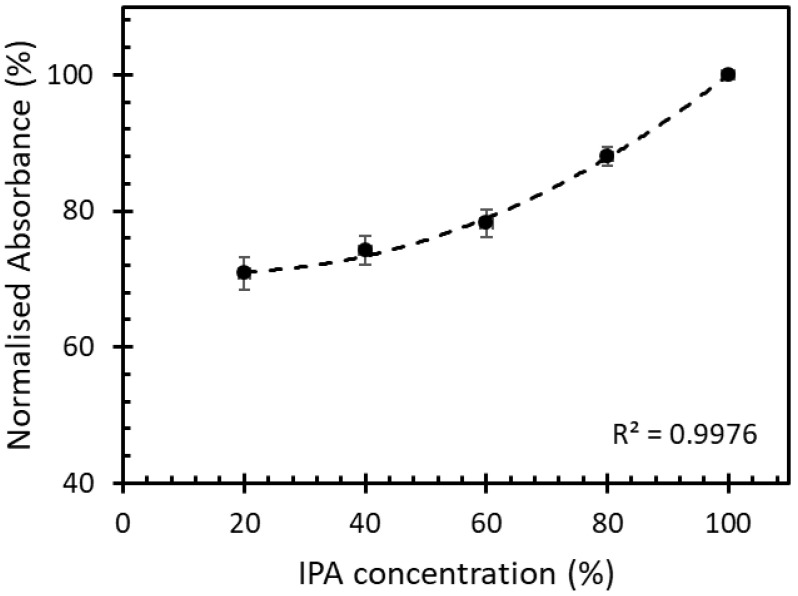
Normalized absorbance of IPA vapor in free space with different concentrations at a fixed time.

The IPA solutions with 20%, 40%, 60%, 80%, and 100% volume fraction were left to evaporate inside the chamber at room temperature. The different concentrations of evaporated IPA in free space were studied for its absorbance at a fixed time by using the UV-VIS spectrometer (PerkinElmer, Waltham, Lambda850+, MA, USA). The absorbance of different IPA concentrations is shown in Figure 6.

At a fixed time, the absorbance of the IPA vapor in free space with different concentrations ranging between 20% and 100% had a 2nd-degree polynomial increment with the correlation of 0.99. This kinetic absorption of the IPA vapor was used to study the absorption of the IPA vapor on the sensor studied in this work.

## 3. Results and Discussion

### 3.1. Saturation Time

**Figure 7:**
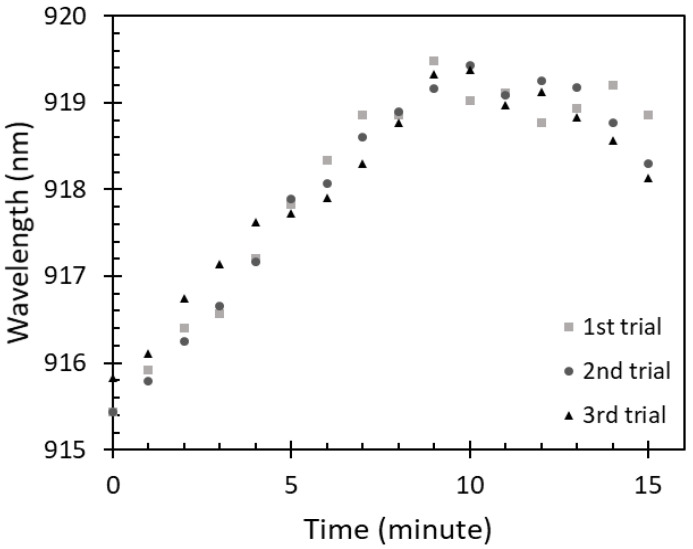
Saturation of 100% IPA vapor absorption on the fiber sensor.

To study the saturation of the IPA vapor absorbed on the sensing region of the optical fiber sensor, the sensor was exposed to 100% IPA vapor concentration for 15 min to observe possible changes of the peak wavelength over time. A relation between the exposure time of the sensor to 100% IPA vapor concentration and the peak wavelength is shown in Figure 7.

It can be observed that the peak wavelength was significantly red shifted in the first 9 min, dropping slightly thereafter where the changes were insignificant compared with what was recorded for the first 9 min. The changes in the first 9 min occurred due to the increase of IPA vapor absorption on the ZnO layer. Therefore, the saturation time of 100% IPA vapor absorption on this sensor was considered to be around 9 min. Similarly, to the free-space absorbance of IPA vapor, an amount of IPA vapor absorbed on the sensor can be varied according to the IPA concentrations at a fixed time. With a lower IPA concentration, i.e., 80%, 60%, 40%, and 20%, a lower amount of IPA absorption on the sensor could be observed due to a smaller volume of IPA evaporation. This resulted in the wavelength shift. Therefore, to investigate the sensor adsorption characteristics for the lower concentrations of IPA vapor, the sensor was exposed to the IPA vapor at a fixed time of 9 min.

### 3.2. Sensitivity Measurement

**Figure 8:**
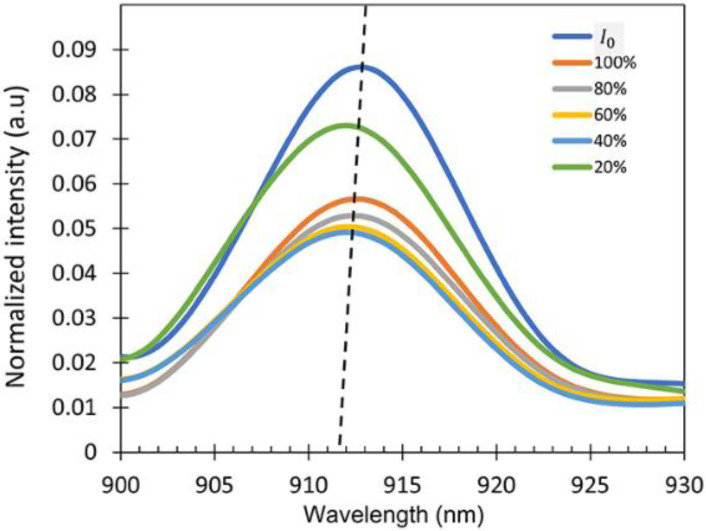
Intensity spectra at different IPA concentrations showing peak wavelength at around λ = 900–930 nm (dotted lines showing the shift as a guide to the eye).

To check the sensitivity of the device, the intensity spectra of the output signal (I) exposed to the IPA vapor for different IPA concentrations varying from 20% to 100% were measured as shown in Figure 8. A wavelength interrogation was studied as the sensing mechanism for this work because the sensor shows a varying wavelength shift for different concentrations of the IPA vapor. It is clearly observed that there was a wavelength shift in the spectrum when the sensor was exposed to the different concentrations of IPA vapor due to the refractive index differences. The intensity of the output signal can be varied due to several factors such as the vibration and bending ratio. No changes in the spectral pattern were observed regardless of the intensity variations in the presence of different concentrations of IPA in the atmosphere, resulting in the consistent wavelength shift in the experiment.

In Figure 7, all the measurements were conducted for 9 min time as the testing was based on the kinetics absorption of IPA on the sensor surface. Therefore, the saturation time for the highest concentration of IPA vapor absorbed on the sensor was used. Transmission spectra were then calculated from the following Equation (7) to accurately observe the wavelength shift compared with the reference intensity ($I_{0}$).

(7) $$T=\frac{I}{I_{0}}$$

where the $I_{0}$ can obtained when the sensor was exposed to the DI water vapor (0% IPA vapor). The sensor exposed to the water vapor was used as the reference to minimize the effect caused by the water evaporation. I is the transmitted intensity when the sensor was exposed to IPA vapor with the concentrations varying from 20% to 100%.

**Figure 9:**
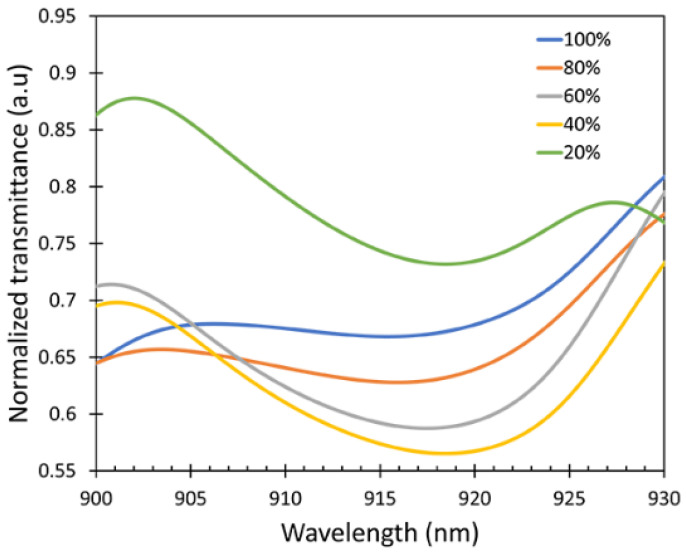
Transmittance spectra peak shifts of the fiber sensor.

The transmittance spectra are shown in Figure 9. From the transmission spectra, it can be observed that the wavelength shifts occurred at λ = 915–920 nm. The response characteristics of the fabricated fiber sensor were investigated at room temperature three times. After each measurement, the sensor was purged with N_2_ gas to remove the IPA absorbed on the ZnO layer and the test cell.

**Figure 10:**
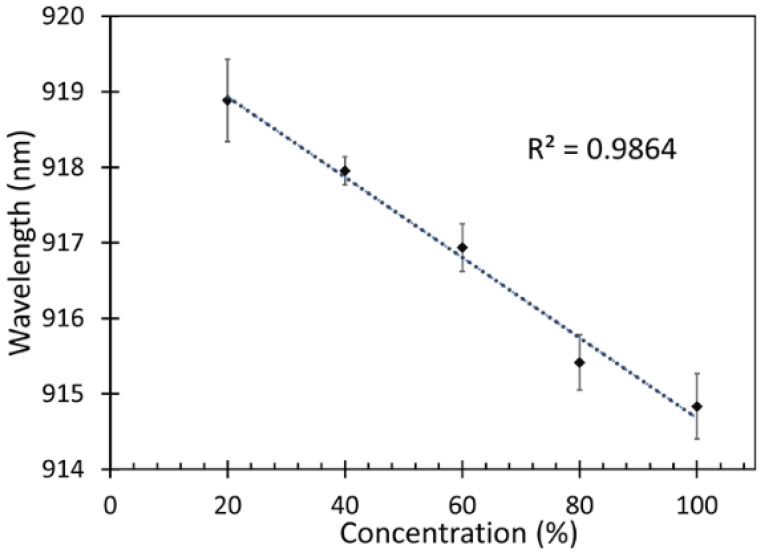
Sensitivity of the fabricated fiber sensor.

After measuring the response of the fabricated fiber sensor, which is presented as a shift in peak wavelength, the correlation between the different concentrations of the IPA vapor and peak wavelength of the fabricated fiber sensor were analyzed for determining the sensitivity of the device as shown in Figure 10.

From Figure 10, it is observed that a blue shift occurred when the sensor was exposed to higher concentrations of IPA. It can also be noticed that the relation between the IPA vapor concentrations and the peak wavelengths was linear with a regression (*r*^2^) of 0.986. Therefore, the sensitivity of the fabricated fiber sensor was defined as a change in the wavelength shift with respect to the IPA concentration. The sensitivity of the fabricated fiber sensor can thus be estimated from the following Equation (8),

(8) $$Sensitivity=\frac{\Delta Wavelength}{\Delta Concentration}$$

The sensitivity of this fiber sensor was found to be 0.053 nm/%IPA vapor. The response time is the time taken for the sensor to react at given concentration of IPA. In this work, the sensor was able to detect changes in IPA concentrations starting from 1 min as shown in Figure 10. However, there was an increase in the absorption of VOC on the ZnO nanorods from 1 min until 9 min, which is the saturation time wherefrom the changes of the sensor response are insignificant. The highest sensitivity was obtained at 9 min, which was chosen to be the response time in this work.

The alcohol vapor adsorption caused a change in the effective refractive index of the coating on the outer cladding, leading to more nanorod–light interaction at the evanescent field of the MMF/CSF region which also triggers the sensing mechanism. This demonstrates that the fabricated sensor was capable of detecting IPA vapor at different concentrations, which is promising. Table 1 shows the comparative study in different research works and our current work.

The sensitivity of our sensor is comparable to the available literature in terms of the similarly fabricated sensor structures, i.e., ZnO on glass substrate, ZnO on SMS fiber, and ZnO doping and composite structures, which is presented in Table 1. The sensitivity of the sensor made of glass substrate coated with ZnO nanorods by Yusof et al. [40] was separated in three different regions: RH between 35% and 55% = −4.4 mV/%; RH between 55% and 70% = −10 mV/%; RH between 70 % and 90% = 24.6 mV/%. The sensitivity of the sensor developed by ZnO thin film coated on MMF in ref. [22] was found to be 0.06 nm/%RH and −0.056 for 50% of C_2_H_5_OH solution and −0.065 for 62% of C_2_H_5_OH solution. Moreover, the sensitivity of the sensor developed by ZnO–CdO composites on alumina tubes with Au electrodes and platinum wires in ref. [41] was found to be 0.153C_IPA_ at temperature 248 °C. Furthermore, the response of 23.6 and 4.7 for 5 ppm and 250 ppb concentrations of IPA were found for Fe-doped ZNO nanoneedles [42]. In our work, the performance of the ZnO nanorods functionalized on the MMF/CSF region showed a sensitivity of 0.053 nm/%IPA, which is comparable to the reported data in the literature. To the best of our knowledge, adequate chemical sensing in the form of vapor concentration is unexplored for similar type of the fiber structure. The sensitivity of our fabricated fiber sensor can be further improved for the detection of low concentrations of IPA by (i) varying the volume fraction of ZnO and the density of ZnO nanorods, (ii) tapering the MMF region to increase the probability of the evanescent field at the sensing region, and (iii) depositing gold nanoparticles on the sensing area to introduce the surface plasmon resonance phenomenon. Therefore, this proposed sensor is considered to have a high potential for further improvement in detecting VOC biomarkers.

## 4. Conclusions

To summarize, a ZnO nanorod coated on a cascaded SMS optical fiber structure was successfully fabricated and implemented for the detection of VOC biomarkers, which is one of the biomarkers targeted for non-invasive diabetes diagnosis. The optical simulation showed a maximum coupling efficiency of the sensing region could be obtained for a sensor length of 9.5 cm with 0.05 volume fraction of ZnO at around λ = 958–980 nm. Hydrothermally grown ZnO nanorods were functionalized on 9.4 cm-long SMS sensors to achieve the maximum possible sensitivity. ZnO nanorods can absorb IPA vapor, which improves the light interaction at the sensor region. The intensity spectra of the fabricated fiber sensors were recorded using an optical spectrometer. The output spectrum showed a peak wavelength that was absorbed by the SMS fiber sensor with the presence of IPA vapor. The fabricated fiber sensor was tested with different concentrations of IPA vapor, i.e., 20%, 40%, 60%, 80%, and 100%. The relationship between the peak wavelengths and IPA vapor concentrations showed a liner trend with *r*^2^ = 0.9864. The obtained results indicated a blue shift at 915 nm to 920 nm wavelength range. The fiber sensor exhibited a sensitivity of 0.053 nm/%IPA vapor, demonstrating the potential of detecting IPA of different concentrations. Hence, the fabricated fiber sensor showed promise to further detect the IPA in exhaled breath, which is one of the VOC biomarkers for diabetes. For the selectivity and sensitivity of the sensor, surface functionalization can be further utilized by coating of gold nanoparticles together with ZnO nanorods and tapering of multimode fibers.

## Figures and Tables

**Table 1:** Comparison of ZnO nanostructures coated on different substrates for alcohol sensing.

Structures	Parameter for Detection	Sensitivity	References
Glass substrate + ZnO-coated	Relative humidity	RH from 35% to 55% = −4.4 mV/% RH from 55% and 70% = 10 mV/% RH between 70% and 90% = 24.6 mV/%	[40]
SMS + ZnO-coated	Relative humidity	0.06 nm/%RH	[22]
SMS + ZnO-coated	Ethanol concentrations	50% of C_2_H_5_OH = −0.056 fitted response curve 60% of C_2_H_5_OH = −0.065 fitted response curve	[22]
ZnO-CdO composites on alumina tubes with Au electrodes and platinum wires	Isopropanol concentrations	0.153C_IPA_ C_IPA_ = gas concentration of IPA at Temperature = 248 °C	[41]
Fe-doped ZnO nanoneedles	Isopropanol concentrations	5 ppm IPA, response = 23.6 250 ppb IPA, response = 4.7	[42]
SMS + ZnO-coated	Isopropanol concentrations	0.053 nm/%IPA vapor (20%–100%) IPA vapor	Present work

## Data Availability

Not applicable.
